# Effect of self-care counselling on depression and anxiety in women with endometriosis: a randomized controlled trial

**DOI:** 10.1186/s12888-020-02795-7

**Published:** 2020-07-29

**Authors:** Nooshin Farshi, Shirin Hasanpour, Mojgan Mirghafourvand, Khalil Esmaeilpour

**Affiliations:** 1 Student Research Committee, Midwifery Department, Nursing and Midwifery Faculty, Tabriz University of Medical Sciences, Tabriz, Iran; 2 Women’s Reproductive Health Research Center, Tabriz University of Medical Sciences, Tabriz, Iran; 3 Midwifery Department, Social Determinants of Health Research Center, Tabriz University of Medical Sciences, Tabriz, Iran; 4 Faculty of Education and Psychology, University of Tabriz, Tabriz, Iran

**Keywords:** Self-care counselling, Anxiety, Depression, Quality of life, Endometriosis

## Abstract

**Background:**

Considering the prevalence of endometriosis and consequent depression and anxiety as well as the resultant effects on the body, mind, and quality of life of patients, this study aimed to determine the effects of self-care counselling on depression and anxiety (primary outcome) and on quality of life (secondary outcome) among women with endometriosis.

**Method:**

This randomized controlled clinical trial was conducted on 76 women with endometriosis who were treated at Al-Zahra Teaching and Treatment Center of Tabriz within the 2015–2019 period. The random blocking method was employed to divide the patients into intervention (counselling) and control groups. In the intervention group, seven self-care group counselling sessions were held on a weekly basis. The control group received routine care. A sociodemographic questionnaire, *Beck Depression Inventory*, *Spielberger State-Trait Anxiety Inventory (STAI)* and the *SF-36 Quality of Life Questionnaire* were completed by the researcher through an interview before and 4 weeks after the intervention.

**Results:**

There was no significant difference between the intervention and control groups in terms of sociodemographic characteristics (*p* > 0.05). After the intervention, the mean scores of state anxiety (mean difference: − 0.12, 95% confidence interval: − 9.6 to − 14.4, *p* < 0.001) and trait anxiety (mean difference: − 10.9: 95% confidence interval: − 9.1 to − 12.7, *p* = 0.001) were significantly lower in the counselling group than those of the control group. The mean score of depression was lower in the counselling group than in the control group; however, it was not significant (*p* = 0/565). The mean score of quality of life for physical health (mean difference = 17.2, 95% confidence interval: 13.8 to 20.5, *p* < 0.001) and for mental health (mean difference = 12.0, 95% confidence interval: 9.0 to 14.9, *p* < 0.001) were significantly higher in the counselling group than in the control group.

**Conclusion:**

Self-care counselling affects the anxiety and quality of life of women with endometriosis. Therefore, in addition to other therapies, this method is proposed to improve quality of life and mental health of patients with endometriosis.

**Trial registration:**

IRCT Registration Number: IRCT 20111219008459 N13, registered on February 10, 2019 (https://irct.ir/user/trial/35915).

## Background

Endometriosis is the second most common gynecological disease, defined as a benign, estrogen-dependent inflammatory disease characterized by the presence and growth of endometrial-like glands and stroma outside the uterine cavity [1, 2]. The etiology of this disease is still unknown. However, some theories have been proposed to explain endometriosis pathogenesis including changes in the immune system, celomic metaplasia (transformation of the germinal epithelium into ovarian endometriosis), benign metastasis (spread of endometriosis), and retrograde menstruation [3]. Accumulating evidence indicates that immune cells, adhesion molecules, extracellular matrix metalloproteinase, and pro-inflammatory cytokines activate or alter peritoneal microenvironment and provide the conditions for differentiation, adhesion, proliferation, and survival of ectopic endometrial cells [4–7]. Although the exact incidence of endometriosis is still unknown, approximately 2–10% of women of reproductive age and nearly 50% of women with pelvic pain are linked to endometriosis [8, 9]. Based on the available knowledge, 47% of women referred for infertility problems have endometriosis [10]. Endometriosis was reported in premenarcheal girls [11] and also in 2–5% of postmenopausal women [12].

Symptoms and severity of endometriosis vary in location, spread, and depth of lesions [8, 13]. The most common symptoms are pain, reduced fertility, gastrointestinal and bladder complications, and heavy and prolonged menstrual bleeding [14]. There is currently no satisfactory therapy for endometriosis. The symptoms (e.g. pain) are relieved with either medication or surgery [15]. The endometriosis treatment aims mainly to relieve pain, improve quality of life, prevent recurrent endometriosis, preserve fertility, and reduce anatomical impact [16, 17].

The chronic nature of endometriosis and potential impacts of its symptoms expose patients to the risk of developing mental disorders. Endometriosis-associated infertility has negative impacts on both marital relationships and social activities [18]. Painful sex and sexual dysfunction (consequences of endometriosis) also have adverse effects on the quality of life, marital relationships and self-confidence and can cause mental disorders [19, 20]. Other endometriosis-associated symptoms including dysuria, dysmenorrhea, dyspareunia, and pelvic pain alleviate the mental health of patients [21, 22]. Overlapping impacts of endometriosis on different aspects of life (physical, psychological, reproductive, communicational, and quality of life) of women allow no separate analysis of these impacts [23]. Endometriosis also has negative impacts on job and education [24, 25].

The relationship between endometriosis, depression and anxiety has been shown in different studies, which indicates the importance of pain perception in developing mood disorders in these women [26, 27]. Laganà et al. indicated high levels of psychiatric disorders (especially somatization, depression, and anxiety) in women suffering from endometriosis [28]. The quality of life of many patients with endometriosis is affected by pain, feelings of fertility loss, anger at disease recurrence, and uncertainty about the future due to the need for reoperation or long-term therapies [29]. Pope et al. stated that endometriosis was accompanied with a wide range of mental disorders, especially depression, anxiety, stress, and low quality of life [21]. Depression, anxiety, and quality of life of patients with endometriosis were assessed in a prospective study. The results showed that 86.5% of the patients developed symptoms of depression. They also found a positive relationship between severity of pain and anxiety [30].

Counselling with self-care focus contributes greatly to the treatment of chronic diseases. Recent studies have shown that patient-oriented counselling encourages patients to actively participate in treatment programs and promotes their positive outcomes [31, 32]. Self-care–based counselling helps the patients to control emotions, adhere to the therapy, understand the treatment rationale, improve quality of life, decrease stress, reduce anxiety, feel more secured, and increase life satisfaction [33]. Adequate knowledge of the chronic disease helps patients combat the disease. Therefore, the type and style of counselling for chronic diseases contribute significantly to the treatment of patients [34, 35].

According to the American Society for Reproductive Medicine, endometriosis should be viewed as a chronic disease requiring a life-long personalized management plan [36]. Patients with chronic illnesses are inevitably in charge of their own daily care. Therefore, they can determine the severity of their symptoms and effects of each type of treatment [36, 37]. According to the World Health Organization, self-care is defined as “the ability of individuals, families, and communities to improve health, prevent disease, maintain health, and cope with illness and disability either with or without the support of caretakers”. Self-care is a self-guided active practice required to prevent short-term and long-term complications [38]. It shows that the person is responsible for health-related behavior and activities needed to control and assess individual health [39]. Self-care for chronic diseases helps maintain physical and mental health, reduce mortality rates, decrease healthcare costs, increase patient satisfaction, and improve quality of life [37, 40].

Current non-surgical treatments for endometriosis (e.g. non-steroidal anti-inflammatory drugs, oral contraceptive pills, and hormonal therapies) limit efficacy [41], and approximately 25–50% of patients discontinue treatment due to adverse effects of medication [42]. Therefore, women with endometriosis tend to practice self-care and lifestyle interventions to relieve some of the symptoms or avoid side effects of the medications [43]. Self-care is a conscious self-regulatory learning practice needed to supply and maintain necessary resources in order to preserve physical, mental, social, spiritual function and growth for survival. It is a life-long practice at in all aspects [44].

Given the importance of psychological outcomes of endometriosis and insufficient assessment of impacts of self-care counselling on depression and anxiety in women with endometriosis, the authors decided to conduct a study to determine the impacts of self-care counselling, based on Orem’s model, on depression, anxiety (primary outcome), and quality of life (secondary outcome) in women with endometriosis.

## Method

### Research design and participants

This study is a randomized-controlled trial, based on the CONSORT guideline with single-blinding (the analyzer of outcomes was blinded to the study groups) and two parallel arms with 1:1 allocation ratio. It was conducted on 76 women with endometriosis aged between 15 and 45 years and hospitalized at Al-Zahra Teaching and Treatment Center of Tabriz within the 2015–2019 period. The inclusion criteria were residing in Tabriz, having at least secondary school education degrees, being diagnosed with endometriosis via laparoscopy during the past 5 years, being in the range of 15–45 years age, and being accessible vie fixed phone or cellphone numbers. The exclusion criteria were any condition that increased the risk of anxiety and depression (e.g. irritable bowel syndrome, migraine, and autoimmune disease …), intake of antidepressants in the past 3 months, malignancies according to the patient, severe depression (29 < depression scores< 63) and very severe anxiety (state anxiety scores> 75 and trait anxiety scores> 72), a recent trauma (e.g. death of relatives or divorce) increasing the risk of developing mental disorders, speech or hearing disorders complicating communication with the researcher, being pregnant after being diagnosed with endometriosis, and a history of past mental illness or a history of hospitalization for the same reason.

G-Power was employed to selected 35 participants as the research sample, based on the study by Waller et al. [45], by taking into account the depression variable and the largest standard deviation of depression subscales (m1 = 11.8, m2 = 7.2 with the assumption of 35% reduction in the depression score due to intervention, sd1 = sd2 = 6.6, α = 0.05 and power = 80%). The sample size was determined 38 by taking into account 10% sample loss.

### Sampling and randomization

The participants were selected after the license from the ethics committee of Tabriz University of Medical Sciences was obtained (ethical code: REC.1397, 625 TBZMED.IR) when the study was registered on the Iranian Registry of Clinical Trials (IRCT 20111219008459 N13). The researcher visited Al-Zahra Teaching and Treatment Center of Tabriz, prepared a list of women with endometriosis hospitalized in the center during the past 5 years based on medical records, and contacted the patients via phone calls to explain the research objectives and design. In a face-to-face meeting with eligible patients, the research objectives and design were then thoroughly explained, and informed written consent forms were obtained. The sociodemographic questionnaire, *Beck Depression Inventory (BDI-ΙΙ)*, *Spielberger State-Trait Anxiety Inventory (STAI)*, and the *Quality of Life Questionnaire (SF-36)* were completed in the interviews. The patients with mild to severe anxiety (32–75 state anxiety scores and 32–72 trait anxiety scores) and mild to moderate depression (14–28 depression scores) were enrolled in the study.

The patients were divided into intervention (counselling) and control groups by using stratified block randomization (with blocks of 4 and 6) based on infertility history via the www.random.org website. The intervention was written down on a paper and placed in sequentially numbered opaque sealed envelopes by a person who was not involved in sampling and data analysis in order to conceal the allocation sequence. The envelopes were given to the participants in order of arrival. For stratification based on infertility history, half of the envelopes were allocated to people with a history of infertility, whereas the other half were allocated to people without a history of infertility; therefore, the group type was determined.

### Intervention

For the intervention group, self-care group counselling was conducted based on Orem’s self-care model in seven 60–90-min sessions in the native language on a weekly basis at Al-Zahra Training and Treatment Center in a relaxing and friendly environment. There were 7–8 participants in each session.

Orem’s Self-Care Theory, known as a grand theory, has effectively been used on some of chronic diseases such as diabetes, coronary artery disease, and cancer. Orem’s theory focuses on each individual’s ability to perform self-care, identifying patients’ needs and the role of groups’ structured relationships as well as defined tasks and allocated responsibilities for providing group members with healthcare [46]. Therefore, in this study group, counseling self-care protocol was designed based on general care needs of women with endometriosis and Orem’s self-care model.

### Contents of the sessions are as follows

#### First session

The number and duration of each session, the interval between sessions, the rules governing the sessions, definition and concept of endometriosis, etiology, diagnostic methods, current treatment methods, and complications were explained to the patients in this introduction session. An educational booklet was also distributed among the patients.

#### Second session

The concept and importance of self-care were explained to the patients in a simple tone. Self-care skills and aspects (e.g. physical, psychological, social, and spiritual aspects of self-care) were also paraphrased.

#### Third session

Necessary training was given in proper and healthy diet. It was recommended to eat fresh and organic (natural) food, and their diet should be rich in antioxidants (vitamins A, C, E, etc.), high in fruits and vegetables, low in saturated fats, and rich in anti-inflammatory substances (e.g. broccoli, avocados, flaxseed, and flaxseed oil).

#### Fourth session

The importance of physical exercise in healthy lifestyle and relief of endometriosis symptoms (especially pain) were explained in a simple tone to cover the role of exercise in improving blood flow, releasing endorphin (a hormone that makes the body feel good), lowering estrogen levels, improving sleep, and reducing stress, anxiety and depression.

The proper medication regimen (e.g. timing and amount of medication) was also discussed, and it was recommended that medications should be taken regularly under the supervision of a doctor and that they should never arbitrarily increase, decrease, or stop the use of medications. The key points were repeated at the end of each session.

#### Fifth session

Non-pharmacological pain management therapies (e.g. yoga and massage) were explained, and the factors worsening the endometriosis symptoms and harmful practices were also explained and was recommended to be avoided (e.g. alcohol, caffeine, red meat, fast foods, and processed foods).

#### Sixth session

The self-care aspects and their impacts on physical and mental health were explained to the participants:

Physical self-care includes exercise, healthy diet, adequate sleep, and avoidance of high-risk behavior.

Psychological self-care includes the desire to live, consideration of leisure time, enjoyment of life.

Social self-care includes having social activities, maintaining friendships, and participating in fun activities with family and friends.

Spiritual self-care involves a close relationship with God.

#### Seventh session

In this session, participants’ questions on all areas of self-care were answered, and the ambiguities were removed.

The group facilitator was a member of the research team who was totally aware of the educational content of meetings, was acquainted with counselling rules, and had the necessary skills to involve everyone in the meeting and provide an environment of empathy, intimacy, and respect.

The control group received routine care. The *Beck Depression Inventory*, the *Spielberger Anxiety Inventory*, and *SF-36 Quality of Life Questionnaire* were completed by the researcher through an interview via phone calls 4 weeks after the intervention (12 weeks after the initial assessment).

#### Data collection tools

The abovementioned tools were employed to collect data.

The sociodemographic questionnaire was utilized to collect data on age, marital status, duration of marriage, number of children, history of infertility, history of treatments for infertility, women’s desire to be pregnant again, ethnicity, education, occupation, spouse’s education, spouse’s occupation, adequacy of income to cover life expenses, place of residence, life satisfaction, duration of endometriosis, time of diagnosis, treatment methods, and post-treatment status. This was a researcher-made questionnaire, the content validity of which was confirmed by 10 faculty members of Tabriz University of Medical Sciences.

##### Beck depression inventory

The *Beck Depression Inventory* was developed by Beck et al. in 1996. It contains 21 items that assesses all domains of depression based on the cognitive theory of depression. Each item has four options. The individuals reveal their feelings and behavioral characteristics by answering each question. Each option is scored from 0 to 3 depending on the severity of symptoms. The scores range from 0 to 63. The inventory can be applied to the members of populations aged above 13 because it assesses both incidence and severity of depression. This scale reflects feelings during the past 2 weeks. Scores ranging between 0 and 13 represent minimal depression, whereas 14 < scores< 19 represent mild depression, and 20 < scores< 28 represent moderate depression. Moreover, 29 < scores< 63 represent severe depression [47]. Rajabi et al. assessed reliability of the *Beck Depression Inventory-Second Edition* by calculating Cronbach’s alpha coefficient. Alphas for the whole questionnaire, the first factors (cognitive-affective), and the second factors (negative attitudes-somatic symptoms) were reported as 0.86, 0.84, and 0.87, respectively. These coefficients were acceptable and showed homogeneity of the subscales. The correlation coefficients of the whole scale with the first and second factors were 0.90 and 0.95, respectively. The correlation between the first and second factors was reported as 0.75; these coefficients were significant at *p* < 0.001 [48].

##### State-trait anxiety inventory (STAI)

This is a standard questionnaire, the reliability of which was confirmed in previous studies. The scale used in this study contained 40 self-report items (20 for the state anxiety and 20 for trait anxiety). The state anxiety is scored on a four-point Likert scale (1) very low, (2) low, (3) high, and (4) very high). The trait anxiety is also scored on a four-point Likert scale ((1) almost never, (2) sometimes, (3) often, and (4) almost always). The minimum and maximum scores were 20 and 80, respectively. Items 1, 2, 5, 8, 10, 11, 15, 16, 19, 20, 21, 23, 26, 26, 27, 30, 33, 34, 36, 36, 39 were scored inversely [49]. In state anxiety, 20 < scores< 31 represent mild anxiety, whereas 32 < scores< 53 represent moderate anxiety. Moreover, 54 < scores< 64 represents relatively severe anxiety, whereas 65 < scores< 75 represent severe anxiety, and scores> 75 represent very severe anxiety. In trait anxiety, 20 < scores< 31 represent mild anxiety, whereas 32 < scores< 52 represent moderate anxiety. Furthermore, 53 < scores< 62 represent relatively severe anxiety, whereas 63 < scores< 72 represent severe anxiety, and scores> 72 represent very severe anxiety. Mahram et al. standardized the scale for Iranian population and reported Cranach’s alpha as 0.91 [50].

##### The short form health survey questionnaire (SF-36)

The *SF-36 Quality of Life Questionnaire* is the most popular instrument for measuring the quality of life. It was developed in the United States and translated into different languages. It measures 8 health-related concepts, namely physical functioning (10 items), role limitation due to physical reasons (4 items), physical pain (2 items), public health (5 items), vitality (4 items), social functioning (2 items), role limitations due to emotional reasons (3 items), and mental health (5 items). An item indicating change in public health per year was added to this questionnaire. The score of each dimension was determined by the score of items included in the same dimension. Reliability and validity of the questionnaire was checked by Montazeri et al. in Iran in 2005. They introduced it as a reliable and valid instrument for measuring public health-related quality of life [51].

The reliability of each questionnaire was determined by using test-retest on a sample of 20 women meeting the research eligibility criteria within a two-week interval by calculating Cronbach’s alpha and the intra-class correlation coefficient in the present study. The intra-class correlation coefficients of *Beck Depression Inventory*, *State Anxiety Inventory*, *Trait Anxiety Inventory*, and *SF-36 Quality of Life Questionnaire* were reported as 0.91, 0.88, 0.83, and 0.93, respectively. The Cronbach’s alphas were also reported as 0.87, 0.78, 0.72, and 0.91, respectively.

### Statistical analysis

Data analysis was performed in SPSS 21, and quantitative data normality was determined through the Kolmogorov-Smirnov. All of the variables were normal except the depression. Chi-square, Trend chi-square, Fisher’s exact tests and the independent t-test were conducted to assess homogeneity of the groups in terms of personal and social characteristics. The independent t-test was conducted to compare the mean scores of anxiety, depression and quality of life between the two groups before the intervention. ANCOVA was employed to compare the scores between the two groups within a four-week interval by controlling the baseline values. The Mann-Whitney U test was employed to assess the variables with abnormal distribution. All analyses were based on the intention-to-treat method, in which *P* < 0.05 was considered significance level.

## Result

**Fig. 1:**
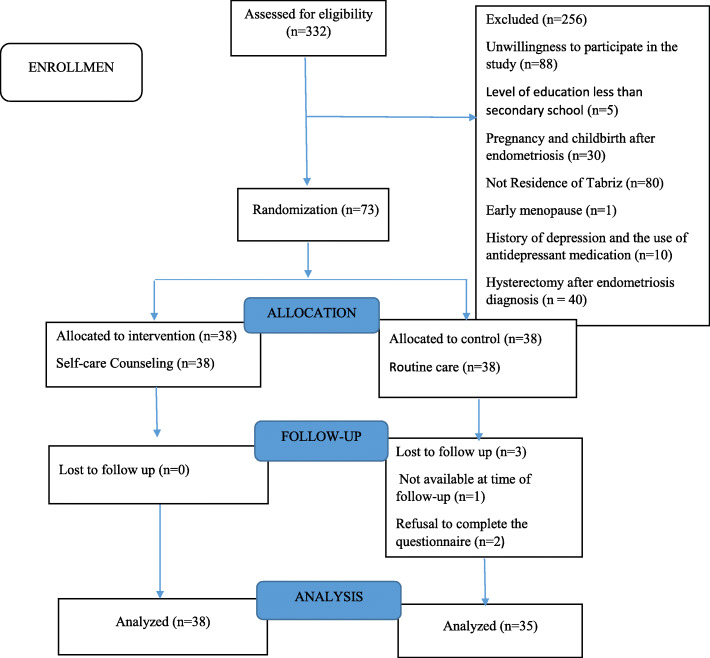
Flow Diagram

There were 332 female participants with endometriosis aged between 15 and 45 years and hospitalized at Al-Zahra Teaching and Treatment Center of Tabriz within the 2015–2019 period. Among them, 256 people failed to meet the inclusion criteria, whereas 76% of them were randomly assigned to the counselling [38] and control [38] groups. Three patients of the control groups left the study (1 person was not available at follow-up time, and 2 people refused to fill out the questionnaires). Therefore, 38 participants in the counselling group and 35 participants in the control group were assessed in follow-up step (Fig. 1).

There was no significant difference between the intervention and control groups in terms of personal social characteristics (Table 1).

**Table 1:** Socio-demographic characteristics of the participants in the study groups

Variable	Counselling group (n = 38) N (%)	Control group (*n* = 38) N (%)	*P*-Value
**Age** (year)^a^	34.8 (6.0)	34.0 (6.2)	0.539^†^
**Marital Status**			0.338^#^
Widow	1 (2.6)	0 (0.0)	
Divorced	5 (13.2)	2 (5.3)	
Married	27 (71.1)	33 (86.8)	
Single	5 (13.2)	3 (7.9)	
**Duration of marriage** (year)^a^	12.9 (6.4)	12.1 (6.9)	0.624^†^
**Number of children**			0.028^#^
No child	11 (28.9)	9 (23.7)	
One child	6 (15.8)	17 (44.7)	
Two and more child	14 (36,8)	8 (21.1)	
**History of infertility**			0.804^‡^
Yes	15 (39.5)	14 (36.8)	
**Willingness to re-pregnancy**			0.715^‡^
Yes	19 (50.0)	19 (50.0)	
**Education**			0.464^§^
Secondary school	12 (31.6)	11 (28.9)	
Diploma	17 (44.8)	15 (39.4)	
University	9 (23.7)	12 (31.6)	
**Job**			0.509^#^
Housewife	28 (73.7)	27 (71.1)	
Employed	10 (26.3)	11 (28.9)	
**Husband’s Education**			0.046^§^
Illiterate	0 (0.0)	1 (2.6)	
Primary school	8 (21.1)	3 (7.9)	
Secondary school	7 (18.4)	5 (13.2)	
Diploma	9 (23.7)	13 (34.2)	
University	5 (13.2)	11 (28.9)	
**Husband’s Job**			0.859^#^
Unemployed	2 (5.3)	1 (2.6)	
Employee	3 (7.9)	6 (15.8)	
Worker	7 (18.4)	9 (23.7)	
Other	17 (44.7)	17 (44.7)	
**Income sufficiency**			0.185^§^
Enough	7 (18.4)	10 (26.3)	
Not enough	12 (31.6)	7 (18.4)	
Relatively enough	19 (50.0)	21 (55.3)	
**House status**			0.904^#^
Personal	19 (50.0)	23 (60.5)	
Rental	10 (26.3)	8 (21.1)	
Woman’s parents’ house	2 (5.3)	2 (5.3)	
Husband’s parents’ house	7 (18.4)	5 (13.2)	
**Satisfaction with marital status**			0.264^§^
Satisfied	22 (57.9)	26 (68.4)	
Relatively satisfied	5 (13.2)	4 (10.5)	
Dissatisfied	2 (5.3)	3 (7.9)	
**Duration of Endometriosis Diagnosis (Year)**^a^	3.9 (2.2)	3.3 (1.9)	0.179^†^
**Treatment**			0.719^#^
Laparoscopy	29 (76.3)	32 (84.2)	
Laparoscopy+ medical	6 (15.8)	5 (13.2)	
Laparoscopy+ medical+ Herbal	2 (5.3)	1 (2.6)	
Laparoscopy+ herbal	1 (2.6)	0 (0.0)	
**Post treatment condition**			0.105^‡^
Recover	32 (84.2)	26 (63.4)	
Recurrence	6 (15.8)	12 (31.6)	

^§^Trend Chi-square test, ^†^ Independent T-test ^#^ Fisher exact test ^‡^ Chi-square test

^a^Numbers are reported in mean (standard deviation)

The median (first and third quartiles) score of depression was 18.0 (14.7–30.0) in the counselling group, whereas it was 17.0 (14.7–25.2) in the control group before the intervention. There was no significant difference between the two groups in terms of depression according to the Mann-Whitney U test (*P* = 0.645). The median (first and third quartiles) score of depression was 12.0 (7.7–23.0) 4 weeks after the intervention in the counselling group, whereas it was 11.0 (7.0–19.0) in the control group. The Mann-Whitney U test results showed no significant difference between the groups in terms of depression by controlling baseline values (*P* = 0.565) (Table 2).

**Table 2:** Comparison of mean depression scores before and 4 weeks after intervention between counselling and control groups

Variable Depression	Counselling group (N = 38) median (first and third quartiles)	Control group (N = 38) median (first and third quartiles)	
Pre intervention	18.0 (14.7–30.0)	17.0 (14.7–25.2)	0.645^a^
Post intervention	12.0 (7.7–23.0)	11.0 (7.0–19.0)	0.565^a^

^a^ Due to the abnormal distribution, the Mann-Whitney U test was used and the median (first and third quartiles) was reported

The depression score range is 0–63

There was no significant difference between the groups in terms of state anxiety before the intervention according to the independent t-test (*p* = 0.507). The mean (standard deviation) of state anxiety score was 44.8 (10.4) before the intervention, whereas it was 35/1(8/0) 4 weeks after the intervention in the counselling group. The mean (standard deviation) of state anxiety score was 43.2 (10.6) before the intervention, whereas it was 47.1 (10.2) 4 weeks after the intervention in the control group. The ANCOVA test results showed significant differences between the two groups in terms of state anxiety score by controlling baseline values (mean difference: − 0.12, 95% confidence interval: − 9.6 to − 14.4, *p* < 0.001). There was no significant difference between the two groups in terms of trait anxiety before the intervention (*P* = 0.136). The mean (standard deviation) score of trait anxiety was 44.9 (9.9) before the intervention, whereas it was 37.0 (8/2) 4 weeks after the intervention in the counselling group. The mean (standard deviation) score of trait anxiety was 41.7 (8.8) before the intervention, whereas it was and 45.6 (9.1) 4 weeks after the intervention in the control group. ANCOVA test results showed significant difference between the two groups in terms of trait anxiety score by controlling baseline values (mean difference: − 10.9: 95% confidence interval: − 9.1 to − 12.7, *p* < 0.001) (Table 3).

**Table 3:** Comparison of mean state and trait anxiety scores before and 4 weeks after intervention between counselling and control groups

Variable	Counselling group	Control group		
	Mean (SD) (*n* = 38)	Mean (SD) (n = 38)	Mean difference (95% confidence interval)	*p-*value
**State anxiety**				
Pre intervention	44.8 (10.4)	43.2 (10.6)	1.6 (−3.2 to 6.4)	0.507
Post intervention	35.1 (8.0)	47.1 (10.2)	−12.0 (−14.4 to −9.6)	<0.001
**Trait anxiety**				
Pre intervention	44.9 (9.9)	41.7 (8.8)	3.2 (−1.0 to 7.5)	0.136
Post intervention	37.0 (8.2)	45.6 (9.1)	−10.9 (−12.7 to −9.1)	<0.001

For comparison of groups before intervention Independent t-test and after intervention, ANCOVA test with controlled baseline values was used

The range of state and trait anxiety scores is 20–80

Table 4 represents the qualitative comparison of depression and anxiety levels before and 4 weeks after intervention between counselling and control groups.

**Table 4:** Qualitative comparison of depression, state and trait anxiety levels before and 4 weeks after intervention between counselling and control groups

Variable	Counselling group n (%)	Control group n (%)	*p-*value^*^
**Depression (**Pre intervention)			
Mild	24 (63.2)	22 (57.9)	0.641
Moderate	14 (36.8)	16 (42.1)	
**Depression (**Post intervention)			
minimal	24 (63.2)	21 (55.3)	0.859
Mild	3 (7.9)	7 (8.4)	
Moderate	11 (28.9)	7 (18.4)	
**State anxiety** (Pre intervention)			
Moderate	29 (76.3)	31 (81.6)	0.553
Relatively severe	7 (18.4)	6 (15.8)	
Severe	2 (5.3)	1 (2.6)	
**State anxiety** (Post intervention)			
Mild	16 (42.1)	1 (2.6)	< 0.001
Moderate	22 (57.9)	25 (65.8)	
Relatively severe	0 (0.0)	6 (15.8)	
Severe	0 (0.0)	3 (7.9)	
**Trait anxiety** (Pre intervention)			
Moderate	30 (78.9)	31 (81.6)	0.735
Relatively severe	7 (18.4)	7 (18.4)	
Severe	1 (2.6)	0 (0.0)	
**Trait anxiety** (Post intervention)			
Mild	14 (36.8)	0 (0.0)	< 0.001
Moderate	22 (57.9)	28 (73.7)	
Relatively severe	2 (5.3)	5 (13.2)	
Severe	0 (0.0)	2 (5.3)	

^*^ Mann-Whitney U test

There was a significant difference between the counselling and control groups in terms of physical health before the intervention according to the independent t-test (*p* < 0.05). The mean (standard deviation) score of physical health was 55.0 (8.1) before the intervention, whereas it was 58.9 (4.9) 4 weeks after the intervention in the counselling group. The mean (standard deviation) score of physical health was 43.8 (6.6) before the intervention, whereas it was 39.5 (5.4) 4 weeks after the intervention in the control group. The ANCOVA test results showed significant differences between the two groups in terms of physical health score by controlling baseline values (mean difference = 17.2, 95% confidence interval: from 13.8 to 20.5, *p* < 0.001). There was a significant difference between the counselling and control groups in terms of mental health before the intervention (*p* < 0.05). The mean (standard deviation) of mental health score was 53.3 (6.2) before the intervention, whereas it was 57.1 (4.8) 4 weeks after the intervention in the counselling group. The mean (standard deviation) of mental health score was 47.5 (7.5) before the intervention, whereas it was 44.7 (4.8) 4 weeks after the intervention in the control group. The ANCOVA test results showed a significant difference between the two groups in terms of mental health score by controlling baseline values (mean difference = 12.0, 95% confidence interval: − 9.0 to 14.9, *p* < 0.001) (Table 5).

**Table 5:** Comparison of mean quality of life scores before and 4 weeks after intervention between counselling and control groups

Variable		Counselling group	Control group		
Quality of life		Mean (SD) (n = 38)	Mean (SD) (n = 38)	Mean difference (95% confidence interval)	*P-*Value
Physical Health Field	Pre intervention	55.0 (8.1)	43.8 (6.6)	11.3 (7.8 to 14.7)	<0.001
	Post intervention	58.9 (4.9)	39.5 (5.4)	17.2 (13.8 to 20.5)	<0.001
Mental health field	Pre intervention	53.3 (6.2)	47.5 (7.5)	5.8 (2.6 to 9.0)	<0.001
	Post intervention	57.1 (4.8)	44.7 (4.8)	12.0 (9.0 to14.9)	<0.001

For comparison of groups before intervention Independent t-test and after intervention, ANCOVA test with controlled baseline values was used

The range of quality of life scores is 0–100

## Discussion

The research results showed a significant reduction in the mean score of anxiety 4 weeks after the intervention in the counselling group in comparison with that of the control group. Furthermore, the mean score of quality of life was significantly higher in the counselling group than in the control group.

Several studies reported that endometriosis was associated with a reduction in some aspects of mental functioning, mental health, and quality of life and that women with endometriosis suffered from depression (86%), moderate to severe anxiety (29%), and mood disorders (68%). The prevalence rates of these disorders were much higher in these patients than in the general population [52]. Since endometriosis is chronic in nature and does not have a specific treatment, controlling its symptoms depends partially on the individual themselves. Apparently, it is essential to design self-care programs.

The effect of self-care counselling on mental health and quality of life in women with endometriosis had not been assessed previously. Therefore, similar studies are reviewed here:

Zhao et al. assessed the impact of progressive muscle relaxation on depression, anxiety, and quality of life in a randomized clinical trial. They examined 100 Chinese women with endometriosis aged between 18 and 48 years undergoing agonist therapy. The therapy lasted for 12 weeks, significantly reduced depression and anxiety, and improved quality of life in the intervention group [17]. The results of their study were consistent with the results of the present study, except for the depression variable. However, the type of intervention and the sample size were different in these two studies.

Alhayek et al. assessed the impact of a certain training program on anxiety and depression in a prospective study in Saudi Arabia. They examined 104 diabetic patients from May 2011 to October 2012. The training program was a videotape regarding diabetes and individual counselling sessions. Depression decreased significantly in the patients after 6 months [53]. The results of their study were inconsistent with the results of the present study. There were no control group and group counselling in the former study, and the target group was not women with endometriosis.

Self-care training courses offer a broad insight into individuality, objective, and life. It encourages people to accept their conditions and act rationally. It guides them to pursue and promote self-care practices [37]. Various aspects of self-care (e.g. diet, physical exercise, and pain control) were discussed in the counselling sessions of the present study which may promote self-control practices and self-care behavior and help the patients to control anxiety. Thus, a significant improvement was observed in women with endometriosis of the counselling group.

There was no significant difference in terms of depression between the counselling and control groups after the intervention in the present study. Counselling did not decrease depression since the chronic endometriosis with no definitive treatment and associated complications (e.g. infertility and stress) significantly increased depression. Probably, the intervention was not efficient enough to resolve many of these tensions.

The results of the present study showed improvement in quality of life after the intervention in the counselling group compared to the control group. The followings are several similar studies in line with the results of the present study in different patients:

A semi experimental study assessed the effects of educational interventions on sexual function and quality of life of 138 women with endometriosis. The intervention improved sexual function and quality of life of these women [54]. The results of this study were consistent with the results of the present study, and the main component of self-care counselling was identified as training and providing necessary information to patients.

A similar semi-experimental study aimed to assess the effect of self-care training on the quality of life of 60 diabetic patients at Seyed Al-Shohada Treatment Center of Tehran. The results showed that proper training on prevention and treatment of diabetes promoted self-care practices, improved public health, and enhanced quality of life of diabetic patients [55]. The results of this study were consistent with the results of the present study. However, the target group and type of intervention were different in the two studies.

Heidari et al. assessed 60 elderly people residing in the Omid Elderly Care Center of Brujen in a controlled randomized trial. They reported that self-care training program for the elderly (proper diet, physical exercise, sleep, and medication) improved their quality of life [56]. Narimani et al. assessed 32 dialysis patients in Maragheh in a semi-experimental study. They reported that self-care training in hemodialysis patients improved their quality of life [57].

To interpret the results, it is fair to state that self-care education improved patients’ information and feelings about the disease and its conditions, the ability to cope with daily life, and the development of self-care behavior promoting inner satisfaction, psychological well-being, and self-efficacy (components of quality of life) [58–60].

Therefore, the present study enriches the research literature on endometriosis by showing that counselling with a self-care approach can improve the anxiety and quality of life among these patients. The patients are eager to acquire information and have self-control on their health problems; therefore, the existence of a multidisciplinary team in managing this disease and integrating self-care counselling programs into the routine care of these patients would be necessary and may promote the health of individuals, families, and societies.

### Limitations and strengths

All participants were literate in this study; therefore, the results cannot be generalized to the population of illiterate women. The research strengths included adherence to all principles of clinical trial (e.g. random allocation and concealment of allocation), completion of the questionnaires by the researcher, and removal of incomplete, false, and ignored items and responses. Moreover, during the counselling sessions, the native language of participants was employed to communicate more easily with them.

## Conclusion

The results showed that self-care counselling might reduce anxiety and improve the quality of life of women with endometriosis. Healthcare providers can use this method of counselling along with other therapies and routine care for women with endometriosis to improve the outcomes of their illness, enhance mental health, and promote the quality of life.

## Data Availability

Data and materials of this study are available from the corresponding author upon reasonable request.

## Abbreviations

BDI: Beck Depression Inventory
STAI: State-Trait Anxiety Inventory
SF-36: Short Form Healthy Survey (36 items)
